# Are predictions of bovine tuberculosis-infected herds unbiased and precise?

**DOI:** 10.1017/S0950268823001553

**Published:** 2023-09-20

**Authors:** Jim Hone

**Affiliations:** Institute for Applied Ecology, University of Canberra, Canberra, ACT, Australia

**Keywords:** bias, bovine tuberculosis, precision, prediction, Zoonoses

## Abstract

Bovine tuberculosis (bTB) is prevalent among livestock and wildlife in many countries including New Zealand (NZ), a country which aims to eradicate bTB by 2055. This study evaluates predictions related to the numbers of livestock herds with bTB in NZ from 2012 to 2021 inclusive using both statistical and mechanistic (causal) modelling. Additionally, this study made predictions for the numbers of infected herds between 2022 and 2059. This study introduces a new graphical method representing the causal criteria of strength of association, such as R^2^, and the consistency of predictions, such as mean squared error. Mechanistic modelling predictions were, on average, more frequently (3 of 4) unbiased than statistical modelling predictions (1 of 4). Additionally, power model predictions were, on average, more frequently (3 of 4) unbiased than exponential model predictions (1 of 4). The mechanistic power model, along with annual updating, had the highest R^2^ and the lowest mean squared error of predictions. It also exhibited the closest approximation to unbiased predictions. Notably, significantly biased predictions were all underestimates. Based on the mechanistic power model, the biological eradication of bTB from New Zealand is predicted to occur after 2055. Disease eradication planning will benefit from annual updating of future predictions.

## Introduction

Bovine tuberculosis (bTB) is prevalent in New Zealand (NZ) and has been the focus of eradication efforts [1–4]. These efforts have been made ongoing for several decades and involve disease testing of livestock (cattle and farmed deer), culling of positive reactors, livestock movement controls, and control of wildlife hosts, especially of brushtail possums (*Trichosurus vulpecula*). Brushtail possums are an important wildlife host of bTB [5–7]. Experimental control measures targeting possums have proven effective in decreasing the incidence of bTB in cattle [8]. In response to the bTB challenge, the broad bTB control approach was revised, and a new strategy was adopted in 2004, which was again revised in 2011 and a new plan was adopted in 2016 [1–4]. The current national aims are to achieve bTB-free livestock by 2026 and bTB-free brushtail possums by 2040 and aim for NZ to be biologically free of bTB by 2055 [7].

Making predictions and evaluating them is a fundamental part of science and can be highly useful in the management of wildlife and their diseases. Predictions generated by bTB models in possums have largely remained untested [9]. Predictions from an earlier study indicated that bTB eradication in NZ could be achievable within 30 years from 2009 [2]. In Britain, predictions regarding breakdowns in bTB control demonstrated high success rates [10]. The validation of predictions in wildlife management studies is encouraged as part of both causal and statistical inference. Many examples have been highlighted in the literature [11, 12]. This study distinguishes between causal inference, which focuses on using logic and evaluating the strength of evidence to determine whether a proposed cause has the observed effect, and statistical inference, which focuses on parameter estimation and inferring population parameter values from a random sample.

The aim of this study is to evaluate the bias and precision of predictions of the number of livestock herds infected with bTB in New Zealand, as part of bTB eradication efforts. These predictions are generated by statistical modelling, which uses trends across years in bTB-infected herds, and mechanistic (causal) modelling, which uses the costs associated with control efforts. These predictions are compared with observed numbers of bTB-infected herds to evaluate bias and precision. The implications for bTB eradication are then described through future predictions.

## Methods

The framework of analyses is analogous to a factorial design with three factors, each having two levels, resulting in a total of eight (2^3^) combinations. The factors are statistical versus mechanistic inference, power versus exponential models, and no updating versus updating of predictions. Statistical modelling focuses on estimating relationships and parameters without specifying any mechanism(s). Trends refer to patterns in diseased herds over time, and since time is not a cause [13], trends do not reveal or infer mechanisms or causes. Mechanistic modelling focuses on evidence of a mechanism(s) generating a pattern. A mechanism is an important aspect of evidence supporting a cause-and-effect relationship [11, 12, 14, 15]. The comparison of predictions not using and those using updating of predictions examines if updating of predictions influences their bias and precision.

Data consisted of the number of livestock herds (cattle and farmed deer) with at least one positive reactor to a bTB test at 30 June each year. It also included the annual costs associated with disease control and vector control published in annual reports of the sequence of agencies responsible for coordinating bTB eradication, which include the Animal Health Board, TB Free NZ, and OSPRI [7]. Disease control costs include expenses related to testing livestock herds, such as bTB skin tests. Vector control costs include expenses incurred in controlling brushtail possums. Total annual costs were calculated by adding of disease and vector costs, and the annual costs were summed starting from and including 1995 to estimate cumulative costs. Other annual costs, as detailed in the annual reports, were not included in the present study. Recent data from 2005 onwards were used, corresponding to the adoption of the new bTB eradication strategy in 2004 extending up to the present year. This period also encompasses a later review in 2011, during which a new objective was developed and adopted [7].

### Statistical modelling of trends

Two models for analysing trends were evaluated: a power equation (H = *a*Y*^b^*) and an exponential equation (H = *a*e^*b*Y^), where H denotes the number of bTB herds and Y denotes year. Additionally, a linear regression which assumed a constant annual change in the number of positive herds, was evaluated, and preliminary analysis showed it was highly biased; hence, it was not examined further. Both the power and exponential models are based on the assumption of diminishing returns. The exponential model assumes a constant proportional change annually. In all analyses involving the power and exponential equations, logarithms with base e were used, either as log_e_-log_e_ or log_e_–linear regression. Furthermore, for each model, the mean squared error (MSE = bias^2^ + variance) [16], was also calculated. Statistical modelling allows a comparison of model predictions with management aims; however, it does not make any inferences about causes.

### Mechanistic (causal) modelling

The relationship between the annual number of bTB herds (H) and the cumulative costs of eradication (C) was examined, assuming a curved relationship, as reported previously [17], and was derived from a cattle–possum bTB model [18]. The power model was analysed using log_e_-log_e_ regression, corresponding to an inverse relationship (H = *a*C*^b^* when *b* < 0) on an arithmetic scale and a linear relationship on a log_e_-log_e_ scale. This power relationship is expected to occur as disease cases, like pest abundance in general, tend to be inversely related to effort (cost) [17, 19]. Additionally, considering the possibility of exponential growth or decline in disease cases [19, 20], an exponential model H = *a*e^*b*C^, was also assessed. In the case of exponential decline, *b* < 0. The costs of eradication were represented by the cumulative costs (NZ$ millions) of possum and livestock disease control, starting from and including 1995. The cumulative costs were used as they provided an overall representation of the combined effects of both current and past controls, without an attempt to separate the effects of each. These costs were not discounted over the years, reflecting a higher value placed on future costs than the case where discounting is applied. In reviews on climate change in the UK [21] and Australia [22], very low discount rates were used, with the latter using discount rates of 1.35% and 2.65%. Previous studies have used discount rates of 7.5%, 8% [2], and 5% [19, 23] when discounting future costs.

### Validation of predictions

In this study, validation refers to the process of comparing observed and predicted values of a parameter. The analysis involved the estimation of a regression relationship, hereafter referred to as the calibration regression, using data from 2005 to 2011 inclusive and then using the out-of-sample [24] data from 2012 to 2021 to evaluate the accuracy of predictions. The predictions were of two types, namely, those derived from the original calibration regression, which projected further into the future progressively, hereafter referred to as no updating. This approach is similar to modelling trends in foot-and-mouth disease (FMD) in the UK in 2001 [25]. The second set of predictions were annually updated by re-estimating the calibration regression each year, resulting in one-year-ahead predictions, hereafter referred to as updated, which is similar to the method used for one-year-ahead predictions of mallard (*Anas platyrhynchos*) abundance in parts of North America [26]. The terms prediction, projection, and forecasting are used interchangeably. The focus is on evaluating quantitative predictions of continuous data, such as abundance, rather than presence/absence data.

The bias and precision of predictions were evaluated using two measures: strength of association (which examines the relationship between predicted and observed data) and difference (which determines whether the observed difference between predicted and expected values includes 0.0, if there is no bias on average). In the association analysis, predicted data were placed on the *y* axis and observed data were placed on the *x* axis [27].

**Figure 1.**
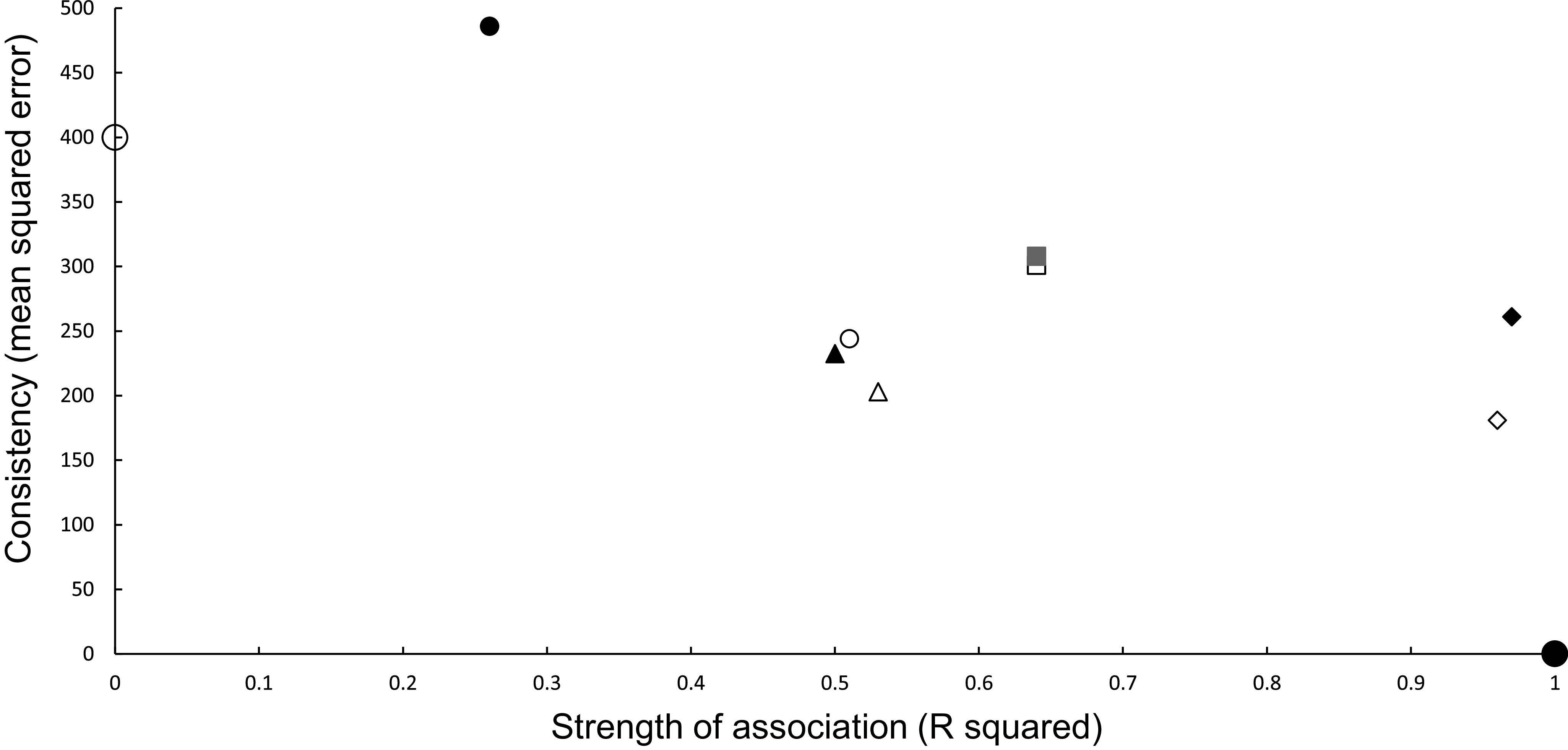
Graphical relationship between causal criteria [14] of strength of association (*x* axis, R^2^) and consistency (*y* axis, mean squared error of predictions). A perfect set of predictions is shown as the large solid circle at (1, 0) and a poor set of predictions as a large open circle (0, 400). Power models are shown as open symbols and exponential models as closed symbols for the same combination of inference and updating. Statistical inferences with no updating are denoted as squares and with updating as small circles. Mechanistic inferences with no updating are represented as diamonds and with updating as triangles. The combination closest to the ideal (large closed circle) uses mechanistic inference and a power model (open diamond).

Causal criteria of strength of association (coefficient of determination R^2^) and consistency [14] (using its inverse measure of mean squared error (MSE)) [16] can be combined graphically (Figure 1). MSE is the vertical (y) axis, and R^2^ is the horizontal (x) axis (Figure 1). A perfect association in a calibration regression has an R^2^ of 1.0 (or 100%), and perfect predictions have no bias and a zero variance, resulting in an MSE of 0. This ideal scenario is a point located at the bottom right corner of the graph at (1,0) (Figure 1). The worst association and predictions have an R^2^ of 0 and a high MSE, positioning them towards the upper left corner of the graph. Multi-model analysis can measure association using Akaike weights which range, like R^2^, between 0 and 1.0 [28], thus can be an alternative measure used on the *x* axis.

## Results

### Statistical modelling of trends: Calibration regression

**Figure 2.**
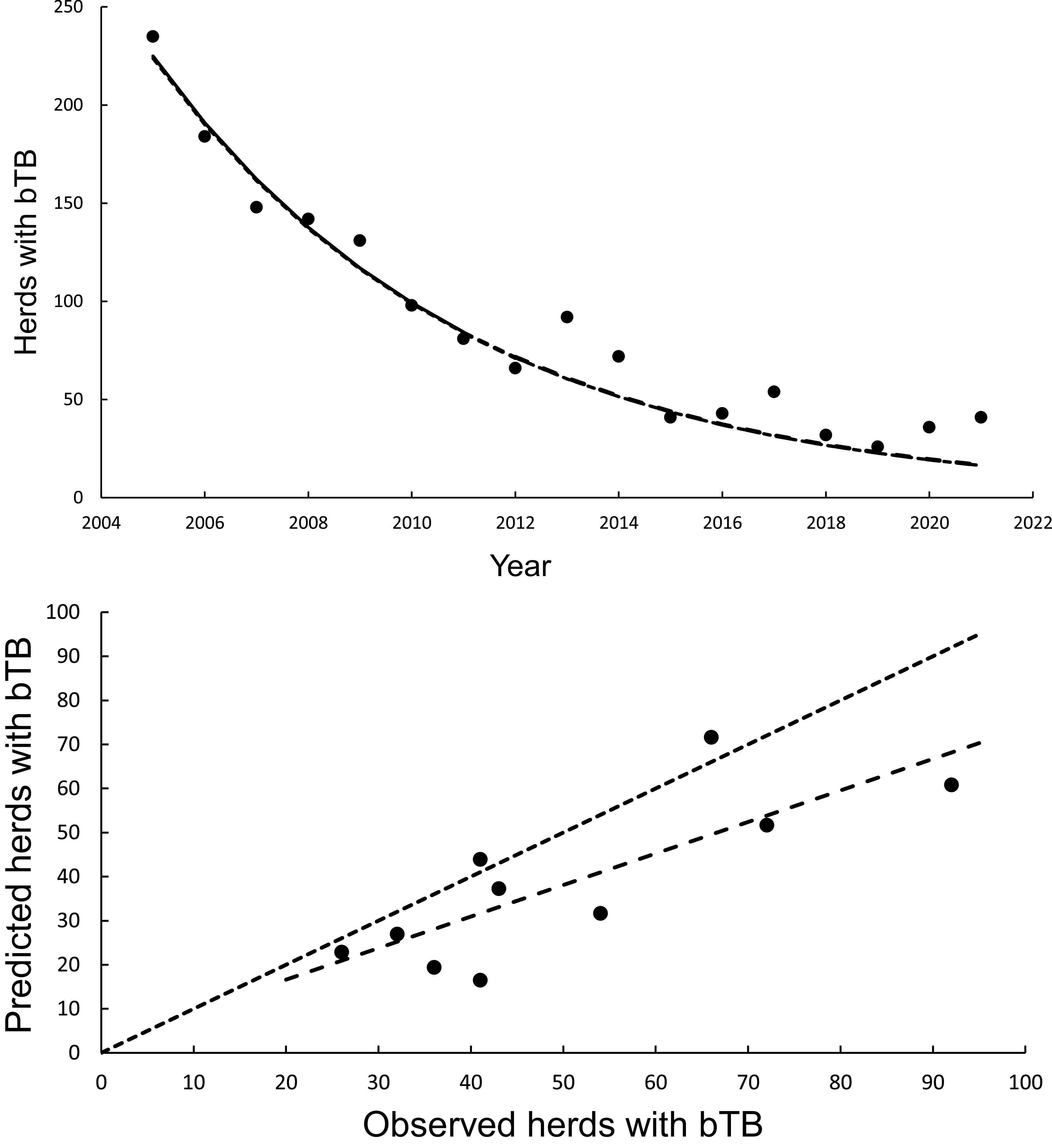
The annual number of herds infected with bovine tuberculosis (bTB) in New Zealand. The data points are shown as solid circles for years 2005 to 2021 inclusive. (a) The solid line is the fitted power model, and the dashed line shows predicted herds with bTB during 2012 to 2021 inclusive. The exponential model is also included in the figure but cannot be seen separately from the power model. (b) Relationships between predicted (*y*) and observed (*x*) numbers of bTB herds. The equality line is shown as the dotted line. Relationships for the power and exponential models are included, although cannot be seen separately.

The curved relationship between bTB herds (H) and years (Y) was strongly supported. The power model on a log_e_H vs log_e_Y scale revealed a significant negative linear relationship (F_1,5_ = 137.00, *P* < 0.0001, R^2^ = 0.97) from 2005 to 2011 inclusive. The estimated slope was −328.259 (95%CI -400.350 to −256.168) and intercept was 2501.300 (95%CI of 1953.056 to 3049.544). After back-transformation, the relationship exhibited a curved, concave-up pattern (Figure 2a). The exponential model on a log_e_H vs Y scale also demonstrated a highly significant linear relationship (F_1,5_ = 137.08, *P* < 0.0005, R^2^ = 0.97), with a slope of −0.1635 (95%CI -0.1994 to −0.1276) and an intercept of 333.1882 (95%CI 261.1155 to 405.2608).

### Statistical modelling of trends: No updating

In the association analyses, the predicted number of bTB-infected herds of the power and exponential models was significantly and positively related (Figure 2b) to the observed number of bTB-infected herds (Table 1). The 95%CI of the estimated slopes included 1.0 and those of the intercepts included 0.0 (Table 1), indicating that both parameters had no bias. However, in the difference analysis, the power model showed a mean bias of 12.0 herds (+/− 4.0 SE), with a 95%CI of 3.0 to 21.0, and a paired *t* value of 3.0 (df = 9, *P* < 0.025), indicating that the power model was significantly biased as it underestimated the number of bTB herds (Table 2). The exponential model exhibited a similar bias (mean = 12.3 herds, +/− 4.0 SE), with a 95%CI of 3.3 to 21.2 (paired t = 3.1, df = 9, *P* < 0.025) (Table 2). The mean squared errors (MSEs) of the power and exponential models were 301 and 308, respectively (Table 3, Figure 1).

**Table 1.** Results of association analyses of the predicted (y) and observed (x) numbers of bTB-infected herds in New Zealand from 2012 to 2021 inclusive

Inference	Statistical		Mechanistic (causal)	
Model	Power	Exponential	Power	Exponential
No updating of predictions				
F_1,8_	14.14	14.14	13.88	14.10
P	0.01	0.01	0.01	0.01
R^2^	0.64	0.64	0.63	0.64
Slope	0.716	0.713	0.501^a^	0.656
LCL	0.277	0.276	0.191	0.253
UCL	1.155	1.151	0.812	1.059
Intercept	2.306	2.156	28.733^b^	6.985
LCL	−21.384	−21.453	11.987	−14.760
UCL	25.996	25.766	45.479	28.730
Annual updating of predictions				
F_1,8_	8.33	2.79	8.93	8.06
P	0.02	0.13	0.02	0.02
R^2^	0.51	0.26	0.53	0.50
Slope	0.564	0.303^a^	0.551^a^	0.524^a^
LCL	0.113	−0.116	0.126	0.098
UCL	1.015	0.721	0.976	0.950
Intercept	16.126	22.143	23.830^b^	19.428
LCL	−8.195	−0.432	0.882	−3.547
UCL	40.447	44.718	46.777	42.404

*Note*: Analyses use statistical inference of trends in bTB herds over the years and mechanistic inference of bTB herds relative to costs. For each form of inference, there are two models (power and exponential).

Abbreviations: LCL, lower limit of 95%CI; UCL, upper limit of 95%CI.

a negatively biased.

b positively biased.

**Table 2.** Mean bias in difference analyses using statistical and mechanistic (causal) inference for each of power and exponential models with no updating and with annual updating of predictions

Inference	Statistical		Mechanistic (causal)	
Model	Power	Exponential	Power	Exponential
No updating of predictions	12*	12*	−4	10*
	(+/− 4)	(+/− 4)	(+/− 4)	(+/− 4)
Annual updating of predictions	6	13*	−1	5
	(+/− 5)	(+/− 6)	(+/− 5)	(+/− 5)

*Note*: Shown are the numerical values of the mean differences (+/− SE) between observed and predicted numbers of bTB-infected herds in New Zealand from 2012 to 2021 inclusive.

* *P* < 0.05 in a paired *t* test. All significant means are underestimates.

**Table 3.** Mean squared errors (MSEs) of the predicted number of bTB-infected herds in New Zealand from 2012 to 2021 inclusive, using statistical and mechanistic (causal) inference for each of power and exponential models with no updating, and with annual updating

Inference	Statistical		Mechanistic (causal)	
Model	Power	Exponential	Power	Exponential
No updating of predictions	301	308	181	261
Annual updating of predictions	244	486	203	233

### Statistical modelling of trends: With annual updating

In the association analysis, one-year-ahead updated predictions of the power model showed a significant and positive relationship with the observed number of bTB herds, with unbiased slope and intercept (Table 1). In the difference analysis, the mean bias (observed – predicted) was 5.8 herds (+/− 4.6 SE), and it was not different from 0.0 (paired t = 1.3, df = 9, *P* > 0.2) (Table 2). The MSE was 244 (Table 3, Figure 1). However, for the one-year-ahead updated predictions of the exponential model, there was no significant relationship with the observed number of bTB herds (Table 1). In the difference analysis, the exponential model provided biased predictions (Table 2). The MSE of the exponential model predictions was 486 (Table 3, Figure 1).

### Statistical modelling of trends: Future predictions

**Figure 3.**
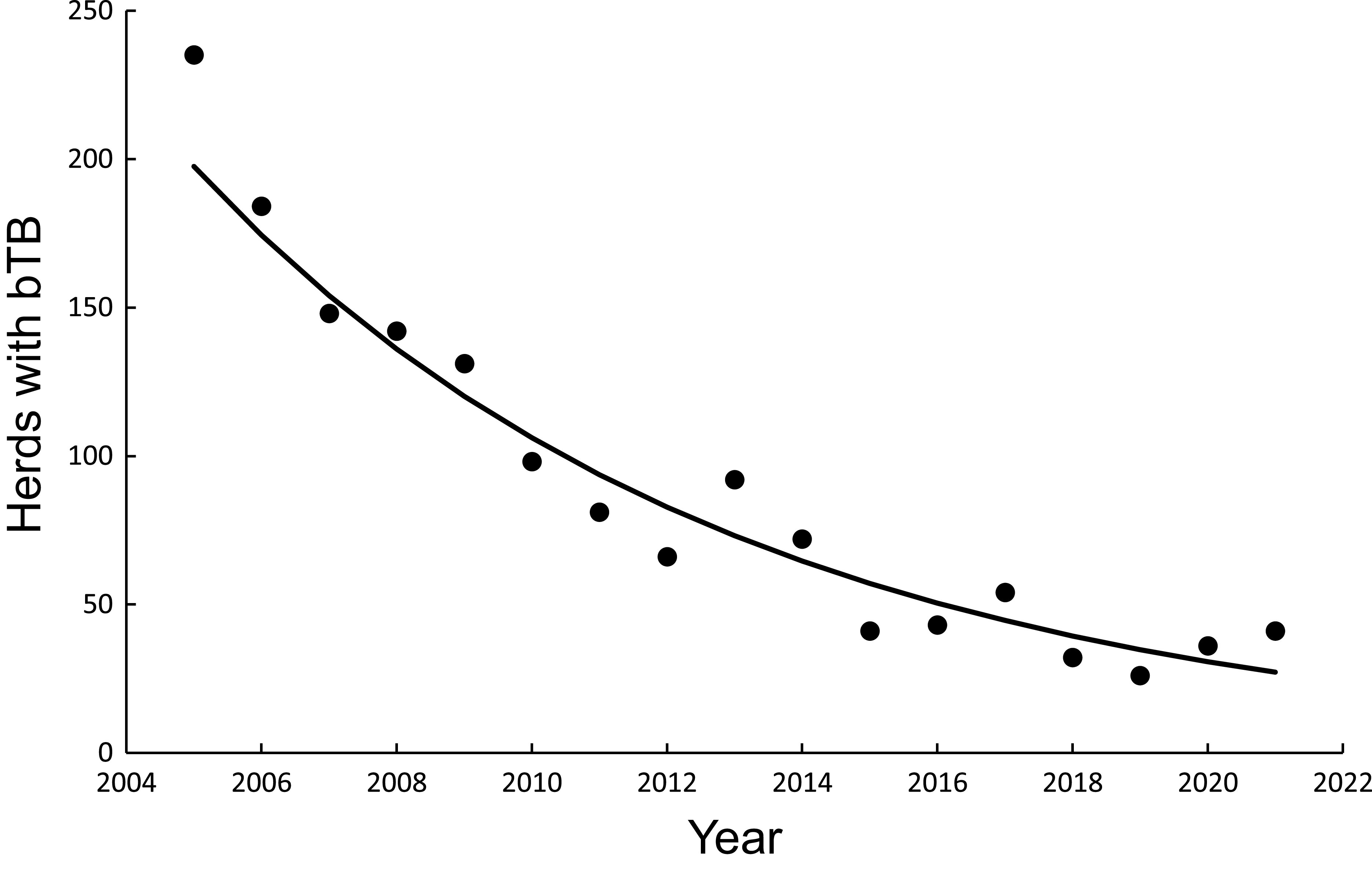
Trends in herds infected with bovine tuberculosis (bTB) in New Zealand and fitted updated power (solid line) and exponential (dashed and dotted line) models, although the fitted lines cannot be seen separately.

There was a significant negative linear relationship (F_1,15_ = 138.10, *P* < 0.0001, R^2^ = 0.90) between number of bTB herds (H) and years (Y) on a log_e_H –log_e_Y scale from 2005 to 2021 inclusive. The power model had a slope of −249.513 (95%CI -294.768 to −204.258) and an intercept of 1902.433 (95%CI 1558.161 to 2246.706). After back-transformation, the relationship displayed a clear curved, concave-up pattern (Figure 3). The exponential model on a log_e_H vs Y scale also demonstrated a similar relationship (F_1,15_ = 137.65, *P* < 0.0001, R^2^ = 0.90), with an estimated slope of −0.124 (95%CI -0.147 to −0.101) and an intercept of 253.766 (95%CI 208.443 to 299.088). Both fitted regressions predicted that the number of positive bTB herds was 24 in 2022, which coincided with the observed number. They also projected a decrease to 3 in the year 2040 and ultimately the eradication of bTB by 2055 (Table 4).

**Table 4.** Predictions by years of the number of herds infected with bTB in New Zealand by statistical inference (trends in herds with bTB by years) and by mechanistic (causal) inference (cumulative costs), for each of the power and exponential models

Inference		Statistical		Mechanistic (causal)	
Year	Aim	Power	Exponential	Power	Exponential
2022	NA	24	24	31	26
2026	Livestock	15	15	24	17
2040	Possums	3	3	12	4
2055	Biological	0	0	7	1
2059	Biological	0	0	6	0

*Note*: The selected years correspond to aims of the national programme of bTB eradication with either or both of livestock and brushtail possums ‘free’ of bTB. The independently observed number of bTB herds in 2022 was 24 [7].

Abbreviation: NA, not applicable.

### Mechanistic modelling: Calibration regression

**Figure 4.**
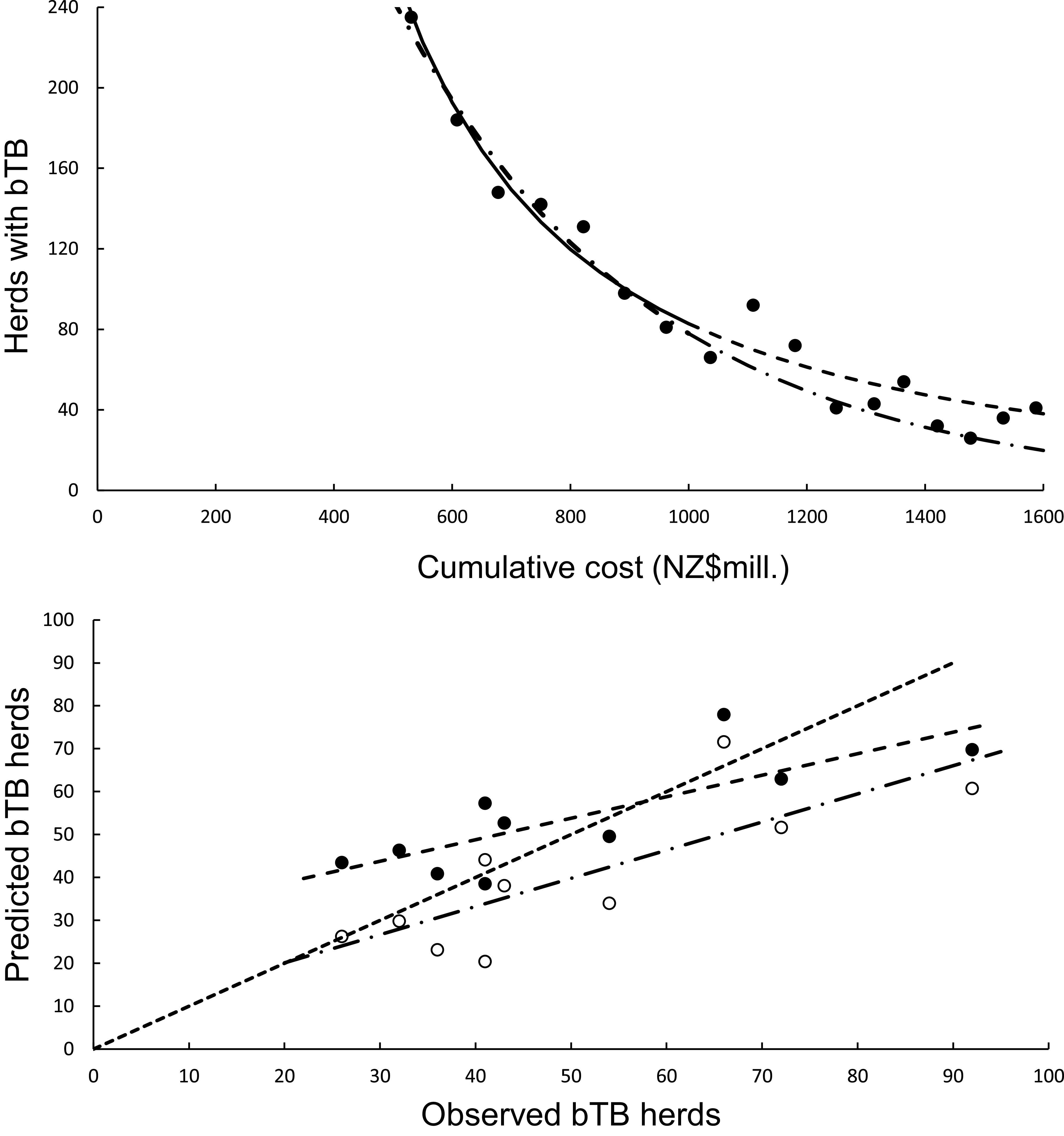
(a) The number of herds infected with bovine tuberculosis (bTB) in New Zealand and the cumulative cost (NZ$ millions) of bTB control starting in 1995. The data points are shown as solid circles for years 2005 to 2021 inclusive, and the solid line is the fitted power function (log_e_-log_e_ regression line) for data from 2005 to 2011 inclusive. The predicted number of bTB herds is shown as a dashed line. The dashed and dotted line is the fitted exponential function, with its corresponding predictions shown as the long dash and dotted line. (b) The significant association between the non-updated predicted (*y*) and observed (*x*) numbers of bTB herds from 2012 to 2021 inclusive. Data for the power model are shown as solid circles with the fitted regression as a dashed line. Data for the exponential model are shown as open circles with the fitted regression as the dashed and dotted line. The dotted line is the equality line.

The curved relationship between bTB herds (H) and cumulative costs (C) was strongly supported. The power model showed a significant negative linear relationship (F_1,5_ = 107.29, *P* = 0.00014, R^2^ = 0.96) between bTB herds (H) and cumulative costs (C) on a log_e_H vs log_e_C scale. After back-transformation, the relationship with predictions was a clear curved, concave-up pattern (Figure 4a). The exponential model showed a significant negative linear relationship (F_1,5_ = 136.230, *P* < 0.00001, R^2^ = 0.97) on a log_e_H vs C scale. After back-transformation, this relationship also displayed a clear curved, concave-up pattern (Figure 4a).

### Mechanistic modelling: With no updating

In the association analysis, the power model showed a significant positive linear relationship between predicted and observed values (Table 1, Figure 4b). However, the slope of the linear regression (0.501, 95%CI 0.191 to 0.812) was less than 1.0, indicating a negative bias, and the intercept (28.733, 95%CI 11.987 to 45.479) was greater than 0.0, indicating a positive bias (Table 1). In the difference analysis, the mean bias of predictions was −3.7 (+/− 4.1 SE), which was not significantly different from 0.0 (paired t = −0.9, df = 9, *P* > 0.20) (Table 2). On the other hand, in the association analysis, the exponential model showed that predicted values were significantly related to the observed values of herds with bTB (Table 1, Figure 4b). The estimated slope was 0.656 (95%CI 0.253 to 1.059), which was not different to 1.0 and hence not biased, and the intercept on the *y* axis was 6.985 (95%CI -14.760 to 28.730), which was not different to 0 and was unbiased (Table 1). However, in the difference analysis, the mean difference between observed and predicted bTB herds was 10.3 herds (+/− 3.9 SE), indicating bias (paired t = 2.6, df = 9, *P* < 0.05), with predictions tending to be, on average, underestimates (Table 2). The mean squared errors of the power and exponential models were 181 and 261, respectively (Table 3, Figure 1).

### Mechanistic modelling: With annual updating

**Figure 5.**
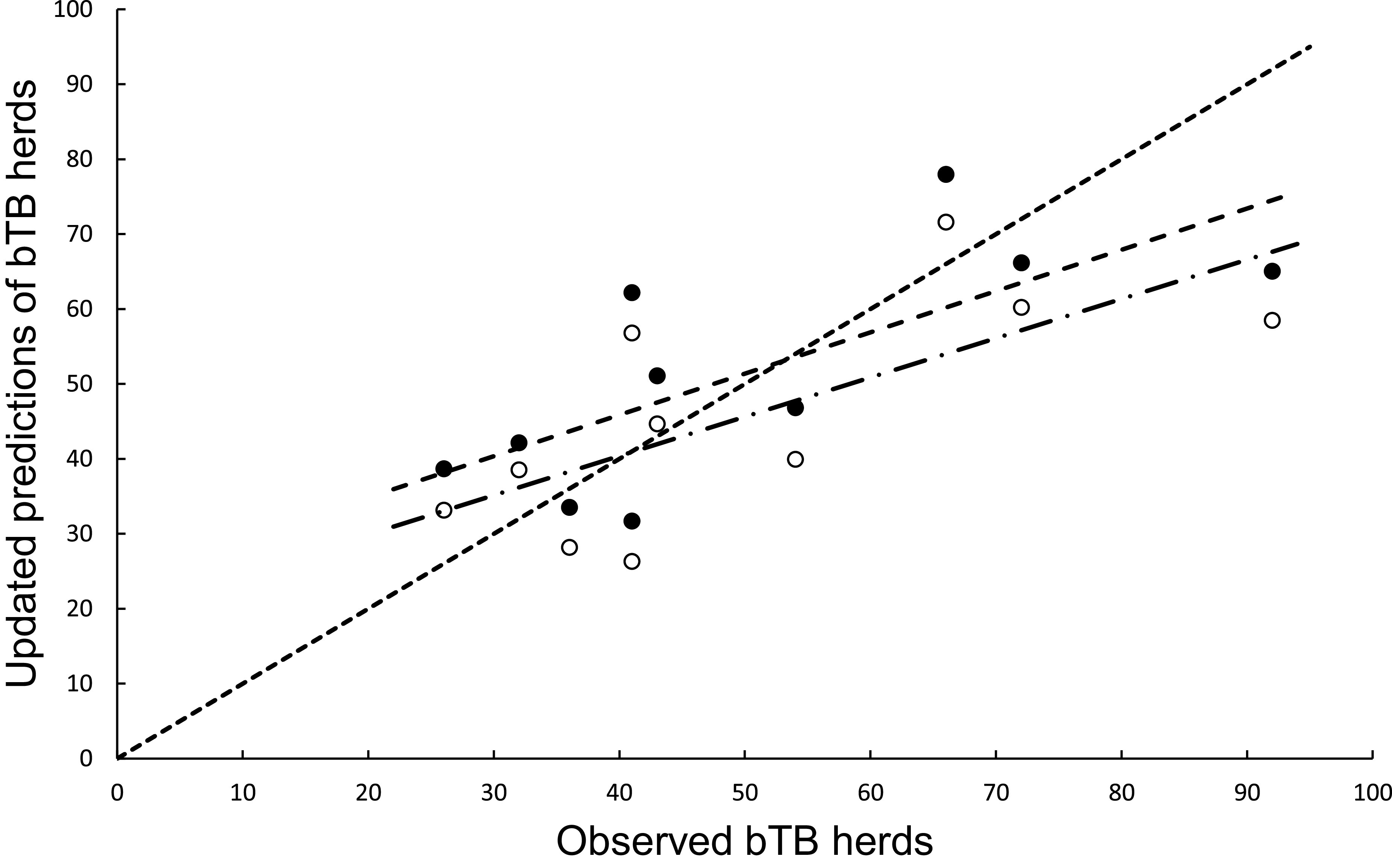
The association between annually updated predictions of the mechanistic power model (solid circles) and observed herds infected with bovine tuberculosis (bTB) and the exponential model (open circles) and observed herds. The dotted line is the equality line, the power model’s fitted linear regression is the dashed line, and the exponential model’s regression is the dashed and dotted line.

**Figure 6.**
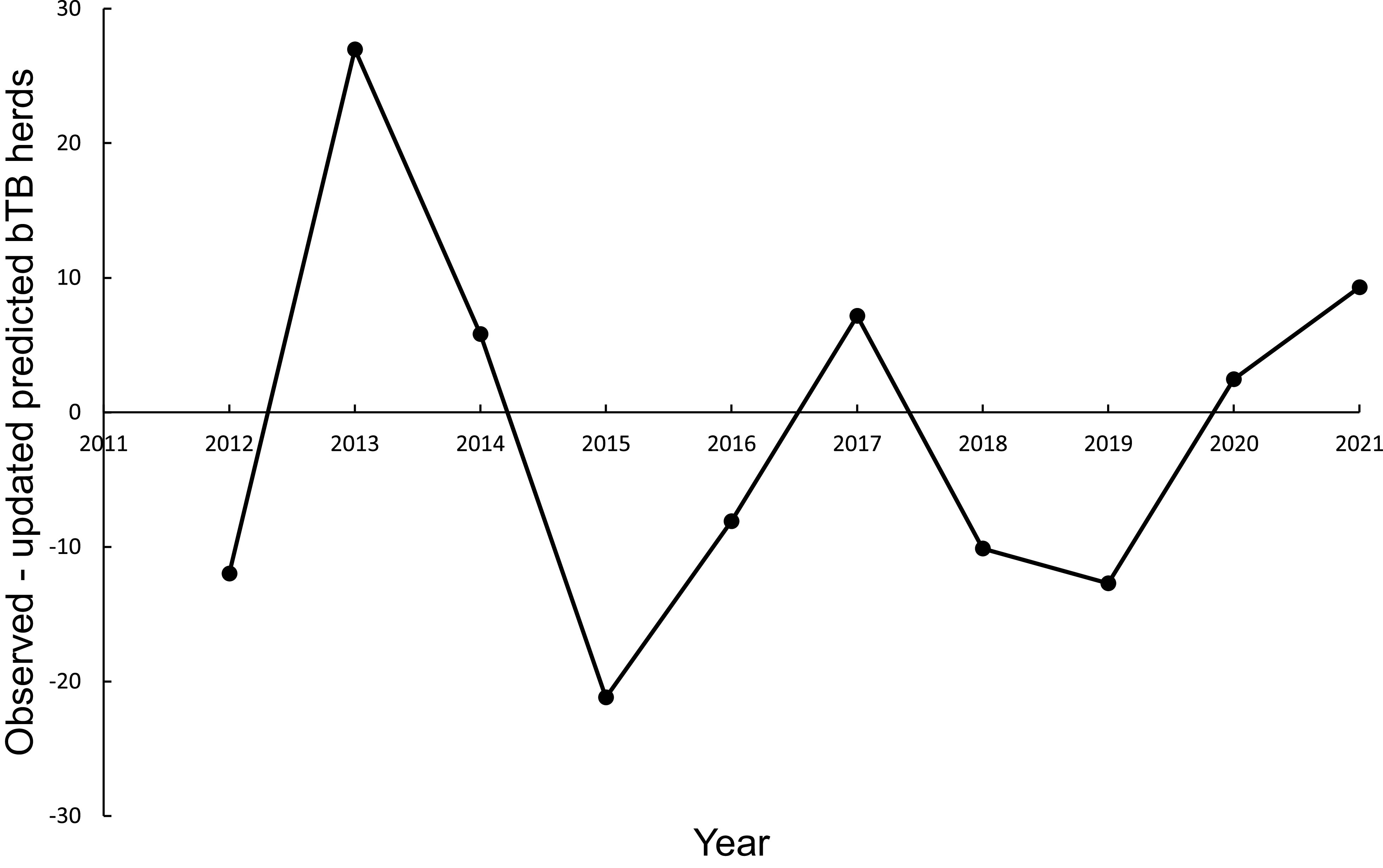
Temporal trends in the differences between the observed number of bovine tuberculosis (bTB)-infected herds in New Zealand and the annually updated number predicted by the mechanistic power model.

In the association analysis, the power model generated one-year-ahead predictions of the number of livestock herds with bTB, and these predictions were positively associated with the observed number of bTB herds (Table 1, Figure 5). However, the fitted linear regression had an estimated slope of 0.551 (95%CI 0.126 to 0.976), indicating a negative bias, and an estimated intercept of 23.830 (95%CI 0.882 to 46.777), suggesting a positive bias (Table 1). In the difference analysis, the mean bias (observed – predicted) was −1.2 herds (+/− 4.5 SE), which was not different from 0.0 (paired t = −0.3, df = 9, *P* > 0.5) (Table 2). The mean squared error of this model was 203 (Table 3, Figure 1). Similarly, in the association analysis, the exponential model provided one-year-ahead predictions that were positively associated with observed herds (Table 1, Figure 5). However, the estimated slope was 0.524 (95%CI 0.098 to 0.950), indicating a negative bias, and the intercept was 19.428 (95%CI -3.547 to 42.404), indicating no bias (Table 1). In the difference analysis, the mean bias was 4.5 herds (+/− 4.6 SE), which was not different from 0.0 (paired t = 1.0, df = 9, *P* > 0.20) (Table 2). The mean squared error was 233 (Table 3, Figure 1). The mechanistic power model with updating shows a temporal trend of damped oscillations in bias (Figure 6). This suggests that differences between the observed number of bTB herds and the predicted number vary over the years, but the amplitude of the differences decreases, approaching zero (Figure 6).

### Mechanistic modelling: Future predictions

Predictions were also made for the future number of herds with bTB using data from 2005 to 2021 inclusive. The power model showed a high level of significance (F_1,15_ = 163.72, *P* < 0.0001, R^2^ = 0.92). The fitted equation is as follows:

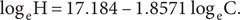

The equation predicted that the number of bTB herds (H) would be 31 in 2022, 24 in 2026, and 7 in 2055 (Table 4), assuming annual costs remain at the current level of NZ$59million. The actual number of bTB herds in 2022 was 24. According to the fitted power equation, there is a marginal benefit of a reduction of 6 bTB herds when the cumulative cost increases from NZ$1,600 million to NZ$1,800 million and a reduction of 2 bTB herds when the cumulative cost increases from NZ$2,800 million to NZ$3,000 million.

The exponential model was highly significant (F_1,15_ = 159.11, *P* < 0.0001, R^2^ = 0.91). The fitted is as follows:

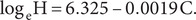



The exponential equation predicted the number of bTB herds (H) would be 26 in 2022, 17 in 2026, and 1 in 2055 (Table 4). The actual number of bTB herds in 2022 was 24. According to the fitted exponential equation, there is a marginal benefit of a reduction of 8 bTB herds when the cumulative cost increases from NZ$1,600 million to NZ$1,800 million and a reduction of 1 bTB herd when the cumulative cost increases from NZ$2,800 million to NZ$3,000 million.

The power model estimates that reducing the number of bTB-infected herds to 1.0 would require a cumulative cost of NZ$10,437 million. On the other hand, the exponential model estimates that achieving the same goal would require a cumulative cost of NZ$3,382 million. In 2021, the cumulative cost was NZ$1,587 million.

## Discussion

The present study has demonstrated that the number of bTB-infected herds in New Zealand (NZ) can be predicted with low bias. Mechanistic models that imply causal relationships received more support than statistical models, and these models described diminishing returns on investments. Specifically, the power model had less biased predictions than the exponential model. Both measures of association and difference yielded some similar findings, but there were also some differences in the results. Future predictions suggest that bTB eradication may take longer than 2055 if the predictions remain unbiased and current strategies and costs of control are maintained.

The control and eradication efforts for bTB in NZ have shown impressive progress [1, 2, 4]. Previous predictions regarding the feasibility of bTB eradication [2] are broadly supported by the findings of the present study, although some differences exist. The current predictions imply that NZ’s livestock will likely be provisionally free of bTB by 2026, which is consistent with the current situation. The statistical models predict biological freedom from bTB by 2055, aligning with the national aims. In contrast, the mechanistic models do not predict biological freedom by 2055 (Table 4), assuming that funding for disease control and possum control remains at current levels. It is worth noting that as the number of bTB-infected herds reaches very low levels, predicting the number may be become difficult because of the very low numbers. Additionally, if strategies change, for example, depopulation of bTB-infected herds and using sentinel animals to detect infection in livestock and wildlife, it may further complicate predictions.

The study does not formally evaluate the aim of achieving bTB-free possums in NZ by 2040 because it is challenging to distinguish the separate effects of concurrent livestock disease control and possum control. Other studies have evaluated various aspects of surveillance and control of possums as bTB hosts [23]. It is noteworthy that Australia eradicated bTB in livestock [29, 30], though Australia did not have a widespread wildlife bTB host, in contrast to possums in NZ. Of the cumulative costs incurred since 2004, 75% (NZ$841.2 m) was spent on possum control and related administration and research, and 25% (NZ$287.4 m) was spent on disease testing and control in livestock. Advancements in disease surveillance, including efforts in wildlife [23, 31, 32], increase the probability of achieving bTB eradication.

Given the substantial costs associated with bTB eradication, it is important to demonstrate benefits [33]. In this study, the clear reduction in bTB-affected herds over the years serves as a demonstrated benefit. It is important to note that the cumulative costs reported here may not be directly comparable to the previously reported results [2]. The curved trends observed in bTB herds in this study (Figure 3) are similar to the curved trends observed in countries with smallpox during the final stages of its eradication [34, Figure 10.4]. A previous study [17] and the current study clearly demonstrate diminishing returns on investments. This pattern of diminishing returns holds implications for other countries engaging in bTB eradication, such as Ireland, which has shown a trend in bTB cases with a possible asymptote above zero, rather than a decline towards zero [35].

A comparison between statistical and mechanistic modelling of climate change revealed that mechanistic modelling yielded less biased results than statistical modelling [36, 37]. Similarly, the results reported here show that mechanistic models produced fewer biased predictions than statistical models (Table 2). The damped oscillations in the bias of the updated mechanistic power model are encouraging and implies increased confidence in the predictions. These damped oscillations are a form of convergence, which has been reported as the correlation between observed and predicted values increasing with larger datasets, suggested as a metric for causal inference [38]. However, correlation alone is not a basis for causal inference. Occurrence of convergence through updating of predictions highlights the importance of annual updating to assess on-going progress in bTB eradication efforts. Annual updating is already established in the management of mallards and their harvest in North America [26]. The utility of validating predictions both for statistical and causal inference has been emphasised in wildlife studies and management [11, 12]. The present study demonstrates that this can be extended to livestock production and disease control.

The study has several limitations. The use of cumulative costs for bTB control may introduce limitations. Firstly, how the same amount of money is spent may differ between years, thus generating extra variation in the *x* variable in the mechanistic models. This variation could reduce the estimated slope of the linear regression between predicted and observed numbers [16] in the association analyses and lead to biased predictions. Secondly, in some analyses, the x variables are not independent as they use cumulative costs. This may increase the risk of a type 1 error (concluding there is a significant relationship when it actually does not exist); however, due to the lack of multiple sites, there was no alternative. Thirdly, this study is observational, rather than experimental, which may lead to weaker causal inferences. Fourthly, the use of bTB numbers from one site across multiple years could provide more precise data than having the same sample size from different sites in a single year. The higher precision might increase the risk of a type 1 error.

In summary, the present study concludes that mechanistic (causal) models provide less biased predictions of the number of bTB-infected herds in New Zealand than statistical models. The results indicate that New Zealand is progressing towards bTB eradication, although it may take longer than the target year of 2055.

## Data Availability

Data are available from the author on request.
